# A Rare Presentation of Small Bowel Diverticulosis Causing Chronic Obstruction and Malnutrition: A Case Study with Review of Literature

**DOI:** 10.1155/2019/2548631

**Published:** 2019-01-13

**Authors:** Y. Mathangasinghe, U. M. J. E. Samaranayake, J. D. Jayasinghe, A. S. K. Banagala

**Affiliations:** ^1^Department of Anatomy, Faculty of Medicine, University of Colombo, Colombo, Sri Lanka; ^2^National Hospital of Sri Lanka, Sri Lanka

## Abstract

Small bowel diverticulosis is an uncommon entity. Clinical presentation of small intestinal diverticulosis is variable. A high mortality is associated with complications such as chronic malnutrition, haemorrhage, intestinal obstruction, and perforation. We report a case of a 63-year-old female with multiple small bowel diverticuli spanning from the first part of the duodenum to the proximal ileum presenting with chronic malnutrition and subacute intestinal obstruction. Although exploratory laparotomy was performed, we opted for a totally conservative treatment in order to avoid complications such as short gut syndrome and anastomotic leakage.

## 1. Introduction

Small bowel diverticulosis is an uncommon entity. Clinical presentation of small intestinal diverticulosis is variable. A high mortality is associated with complications such as chronic malnutrition, haemorrhage, intestinal obstruction, and perforation. We report a case of a 63-year-old female with multiple small bowel diverticuli spanning from the first part of the duodenum to the proximal ileum presenting with chronic malnutrition and subacute intestinal obstruction.

## 2. Case Presentation

A 63-year-old female presented with epigastric pain, loss of appetite, abdominal bloating, regurgitation, and episodic projectile vomiting of five-year duration. These symptoms were aggravated particularly after meals. Her bowel opening was normal. The patient had lost 20 kilograms over five years. The patient had a background history of hypothyroidism for which she was on thyroxine replacement therapy. She was clinically euthyroid. She had undergone a vaginal hysterectomy for uterovaginal prolapse at the age of 39 years. There was no significant family history for bowel disorders.

**Figure 1:**
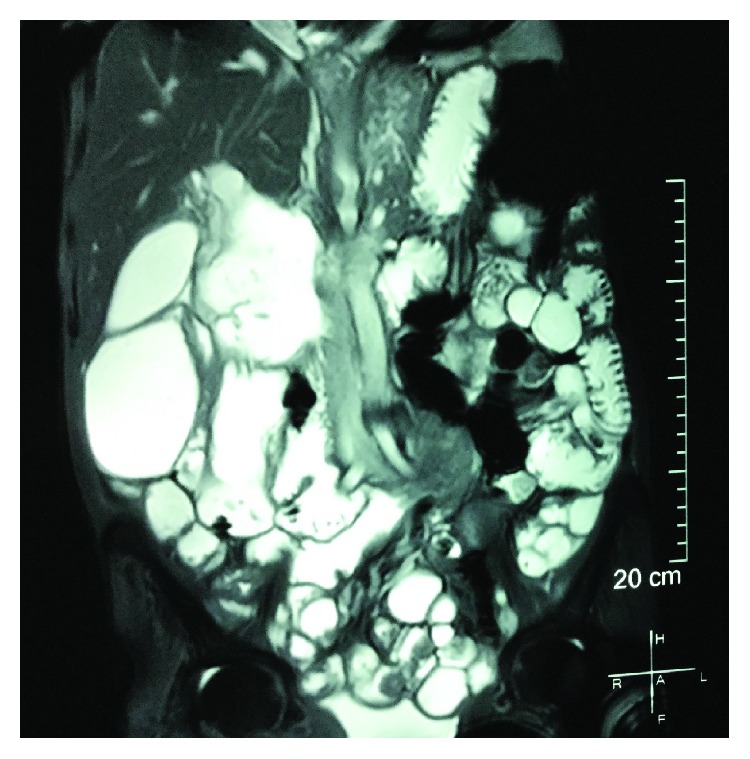
Magnetic resonance enterography showing multiple dilated small bowel loops with loss of valvulae in the right side of the abdomen.

On physical examination, she had a body mass index of 13. She was pale. There were peripheral stigmata of chronic malnutrition and vitamin B12 deficiency. She had a distended abdomen, visible peristalsis, and hyperacute bowel sounds. There was no clinically demonstrable free fluid in the abdomen. She had anaemia (haemoglobin-8.9 g/dl, haematocrit-27.3%, mean corpuscular volume-97.4 fl, mean corpuscular haemoglobin-31.6 pg, mean corpuscular haemoglobin concentration-325 g/l, and red cell distribution width-58.4 fl), with normal platelet (402 × 10^3^/*μ*l) and leucocyte (8.07 × 10^3^/*μ*l) counts. Blood picture showed macrocytic red cells and hypersegmented neutrophils. Abnormal chemical pathological investigations comprised of elevated C-reactive protein (20.1 mg/l), hypoproteinaemia (59 g/dl), hypoalbuminaemia (25.3 g/l), hypovitaminosis B12 (160 pg/ml), and hypocholesterolaemia (total cholesterol-125.5 mg/dl, HDL-32 mg/dl, LDL-66.3 mg/dl, and triglycerides-136.4% with normal VLDL-27.2 mg/dl). Serum ionized calcium was 2.41 mmol/l. Serum iron studies favoured anaemia of chronic disease (serum iron-95 *μ*g/dl, total iron binding capacity-138 *μ*g/dl, iron saturation-40.8%, and ferritin-238 *μ*g/l). She was biochemically euthyroid (TSH-4.65 mIU/l). Ultrasonography was suggestive of subacute small intestinal obstruction with distended first part of the duodenum filled with fluid. Multiple tortuous small bowel loops were noted around the pancreas with increased peristalsis. Large bowel was distended with gas. There was no bowel wall thickening, mass lesions, or free fluid in the abdomen. Computed tomography (CT) of dual slice contiguous axial sections of the abdomen obtained after intravenous, oral, and rectal contrast administrations demonstrated mild dilatation of the first and second parts of the duodenum with no evidence of significant obstruction to distal passage of oral contrast. The stomach was not distended. A focal calcification of the segment VII of the liver was noted, which was likely to be an incidental finding. There was no CT evidence of an annular pancreas or superior mesenteric artery syndrome. A spiral CT was repeated after one week. It showed markedly distended proximal bowel loops involving the duodenum and the proximal jejunum. No definite transition point was identified. There was a whirl appearance seen in the mesentery and superior mesenteric venous branches around the superior mesenteric artery raising the suspicion of a possible midgut volvulus. Mild mesenteric engorgement was also seen. No definite CT evidence of diverticuli was seen. Upper gastrointestinal (UGI) endoscopy revealed multiple duodenal diverticuli with a small hiatus hernia. Barium meal and follow-through study revealed a slightly distended duodenum without evidence of obstruction or persistent narrowing. Magnetic resonance enterography revealed multiple dilated small bowel loops with loss of valvulae in the right side of the abdomen (Figure 1). There were numerous outpouchings arising from the small bowel.

**Figure 2:**
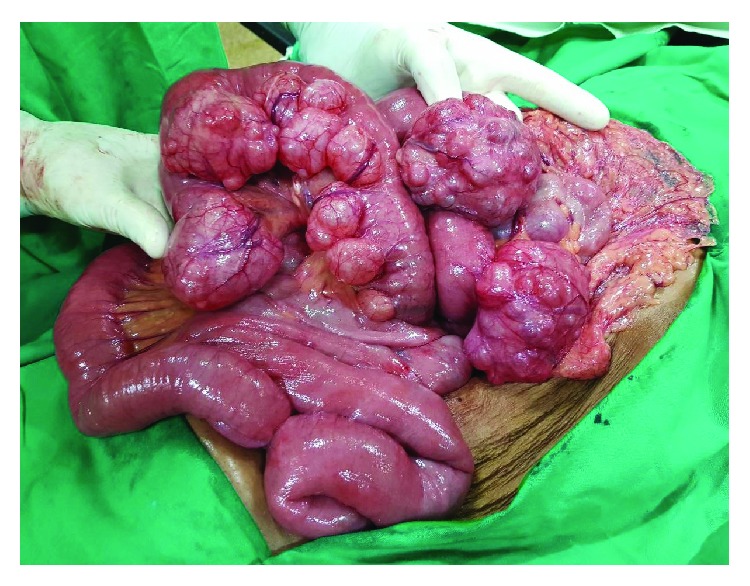
Multiple large diverticuli extending from the first part of the duodenum to the proximal ileum seen during the exploratory laparotomy.

However, with unexplained weight loss, we wanted to exclude a gastrointestinal malignancy and intestinal tuberculosis. We opted for laparotomy out of diagnostic laparoscopy and laparotomy. Multiple large diverticuli were noted extending from the first part of the duodenum to the proximal ileum (Figure 2). Diverticuli were measuring from 0.5-12.0 cm. There was macroscopic evidence of diverticulitis. There was gross gastric dilatation. Proximal small bowel was dilated without a definitive transition point. The rest of the terminal ileum and colon were normal macroscopically. Small bowel was not surgically resected because she was not a suitable candidate for a primary anastomosis as she had nutritional deprivation and the risk of short gut syndrome. We closed the abdomen without any surgical interventions. Because of macroscopic evidence of diverticulitis, intravenous cefuroxime 750 mg 8 hourly and intravenous metronidazole 500 mg 8 hourly were administered for 7 days and were converted to oral cefuroxime 500 mg 12 hourly and oral metronidazole 400 mg 8 hourly for another 21 days. She had an uneventful postoperative period. She received complementary parenteral nutrition with amino acids, electrolytes, dextrose, and lipid injectable emulsions followed by standard polymeric formulae containing whole proteins. Soluble fibres were gradually introduced to her diet. She received a high-calorie diet, initially 125% of the daily calorie requirement followed by 150% of the daily calorie requirement after one month. Thousand international units of vitamin B12 was administered intramuscularly every other day for five days, and oral vitamin B complex 1 mg three times a day was continued for six months, with folate and micronutrient replacement. Iron-rich food and standard formulae were used to supplement micronutrients such as selenium and zinc for six months. She had a remarkable recovery with no recurrence of symptoms following 10 months follow-up.

## 3. Discussion

Multiple small intestinal diverticulosis is an uncommon entity. The jejunum is the commonest site to develop such diverticuli [1, 2]. According to Longo and Vernava, 80% of multiple diverticuli occur in the jejunum, followed by the ileum [3]. The incidence of jejunal diverticulosis is 2% as reported in enteroclysis studies, and it is 2-5% in autopsy studies [4, 5]. The incidence increases with age [4–6]. Up to 60% of patients with small intestinal diverticuli have concomitant colonic diverticula [7–10].

A true intestinal diverticulum is the herniation of mucosal and submucosal layers of the intestine through a defect in the muscular layer [11, 12]. There are discrete entities of small intestinal diverticuli such as Meckel's diverticulum and isolated periampullary lesions [11]. In contrast to true diverticuli, multiple small intestinal diverticuli are considered as submucosal pulsion-type pseudodiverticuli [2, 13]. These are outpouchings at the sites where blood vessels pierce the intestinal wall [12]. Multiple small intestinal diverticuli are congenital or acquired [2]. Majority of the acquired ones are multifactorial. Multiple diverticuli may develop due to intestinal dysmotility. Irregular contractions cause increased intraluminal pressure in different segments of the intestine leading to multiple outpouchings from the sites where the intestinal wall is weak [14]. Multiple diverticuli are known to associate with Cronkhite-Canada syndrome [15], Fabry's disease [16], mitochondrial neurogastrointestinal encephalomyopathy [17], Marfan and Ehlers Danlos syndromes [18, 19], and systemic lupus erythematosis [2]. In contrast to adults, patients with congenital multiple intestinal diverticuli present at a very early age; however, pathogenesis of congenital diverticuli is poorly understood [20].

Majority with multiple small intestinal diverticulosis are asymptomatic [10, 21]. They are known to present with features of malabsorption such as unexplained anaemia, macronutrient and micronutrient deficiencies [1, 22]. Bacterial overgrowth leading to blind loop syndrome is a postulated mechanism of malabsorption [2]. Patients present with chronic abdominal discomfort, which is often postprandial [1, 4, 5, 13]. These multiple diverticuli can get inflamed leading to diverticulitis. Clinical symptoms may mimic gastritis, pancreatitis, or cholelithiasis [11, 23, 24]. Terminal ileal diverticulitis may mimic acute appendicitis [8, 25, 26]. Leucocytosis and elevated inflammatory markers are seen in diverticulitis [8, 27]. Perforation, upper gastrointestinal bleeding, either haematochezia or melaena [10], and intestinal obstruction [1] are known complications of these patients. Intestinal obstruction may be due to dysmotility of the intestine itself or due to an obstructing enterolith [2]. These complications are associated with a high morbidity and mortality [1]. Perforation has a reported mortality between 30% and 40% [21, 26, 28, 29]. Rarely common bile duct obstruction, cholangitis, superior mesenteric vein thrombosis, and duodenal fistula formation are also reported [30].

Multiple small intestinal diverticulosis is often found incidentally in imaging studies [1, 9, 31, 32]. Plain X-rays may demonstrate segmental dilatation of the small intestine. But this sign is neither specific nor sensitive [1]. Pneumoperitoneum on plain X-rays are rarely seen [26, 33–35]. Ultrasonography is less sensitive than computed tomography (CT) in diagnosing this condition [33, 35]. Multislice CT is 81% sensitive and 99% specific [13, 35–37]. Magnetic resonance imaging (MRI) enteroclysis is specific and sensitive in the diagnosis of multiple small intestinal diverticuli presenting as nonemergency cases [13, 38–41]. They reduce the need of emergency laparotomies [13]. CT and MRI findings of small bowel diverticuli include multiple outpouching or air bubbles in the intestine with an inflammatory background [26, 42–44]. UGI contrast studies can be used in the absence of acute diverticulitis [45]. However, UGI barium follow-through studies cannot reliably exclude the presence of small bowel diverticuli as in our case. Maglinte et al. considered that, if the mouth of the diverticulum is large, it can rapidly empty, and therefore, barium will not stay inside the diverticulum to be visualized [46]. This is evident by the high prevalence of small intestinal diverticuli in autopsy series compared to the UGI contrast series [46, 47]. UGI endoscopy can be diagnostic or therapeutic. It can accurately diagnose lesions in the second part of the duodenum. But the diagnostic sensitivity is low since the rest of the bowel is difficult to visualize by UGI endoscopy [48]. Enteroscopy is an emerging technique to diagnose small bowel diverticular disease. Double balloon enteroscopy has shown promise in diagnosing and treating diverticuli in the jejunum causing UGI bleeding [48–50]. Similarly, radioisotope labelled mesenteric angiography is also reported to be sensitive in diagnosing cases which present as occult UGI bleeding [38]. Although double balloon enteroscopy is a promising technique, it was not available for our patient at that time.

Only 1-2% of single duodenal diverticuli [21] and 29% of jejunal diverticuli [10] become symptomatic. Symptomatic uncomplicated diverticuli are managed conservatively [11]. Nutrition modifications are essential in patients who are symptomatic with chronic malabsorption like our case. Symptomatic patients with diverticulitis are also initially attempted to manage conservatively with resting bowel and broad-spectrum antibiotics [13, 36, 51, 52]. Symptomatic patients with bowel perforation due to intestinal diverticuli are usually managed with surgery [53]. However, there are case reports on conservatively managed perforated diverticuli [54]. Up to 30% of complicated diverticulitis with bleeding may need surgical treatment [55]. Surgical resection of the affected segment is the commonest practice in such cases [26, 56, 57]. Excision of only the involved diverticulum is not recommended due to a high rate of complications [8]. Long-term outcome data on patients undergoing intestinal resection are sparse. Recurrence of diverticuli after surgical resection is also reported [1]. Recently, double balloon enteroscopy was used to treat symptomatic patients with bleeding [58]. Similar to our case, laparoscopy was reported as a diagnostic entity for small intestinal diverticulosis [59]. However, apart from laparoscopic resection of solitary small intestinal diverticuli [60], the reports of laparoscopy used as a therapeutic modality for multiple small intestinal diverticuli are sparse.

Nevertheless, there are no randomized controlled trials to compare efficacy and safety of conservative management, double balloon enteroscopy, and elective resections. Similarly, there are no comprehensive guidelines on management of this uncommon entity.
